# Experimental Investigation of Thermal Conductivity of Water-Based Fe_3_O_4_ Nanofluid: An Effect of Ultrasonication Time

**DOI:** 10.3390/nano12121961

**Published:** 2022-06-08

**Authors:** Divya P. Barai, Bharat A. Bhanvase, Gaweł Żyła

**Affiliations:** 1 Department of Chemical Engineering, Laxminarayan Institute of Technology, Rashtrasant Tukadoji Maharaj Nagpur University, Nagpur 440033, MS, India; divyapbarai@yahoo.co.in; 2 Department of Physics and Medical Engineering, Rzeszów University of Technology, 35-959 Rzeszów, Poland

**Keywords:** Fe_3_O_4_ nanofluid, thermal conductivity, ultrasonication time

## Abstract

Nanofluid preparation is a crucial step in view of their thermophysical properties as well as the intended application. This work investigates the influence of ultrasonication duration on the thermal conductivity of Fe${}_{3}$O${}_{4}$ nanofluid. In this work, water-based Fe${}_{3}$O${}_{4}$ nanofluids of various volume concentrations (0.01 and 0.025 vol.%) were prepared and the effect of ultrasonication time (10 to 55 min) on their thermal conductivity was investigated. Ultrasonication, up to a time duration of 40 min, was found to raise the thermal conductivity of Fe${}_{3}$O${}_{4}$ nanofluids, after which it starts to deteriorate. For a nanofluid with a concentration of 0.025 vol.%, the thermal conductivity increased to 0.782 W m${}^{-1}$K${}^{-1}$ from 0.717 W m${}^{-1}$K${}^{-1}$ as the ultrasonication time increased from 10 min to 40 min; however, it further deteriorated to 0.745 W m${}^{-1}$K${}^{-1}$ after a further 15 min increase (up to a total of 55 min) in ultrasonication duration. Thermal conductivity is a strong function of concentration of the nanofluid; however, the optimum ultrasonication time is the same for different nanofluid concentrations.

## 1. Introduction

The dispersion of nanoparticles in base fluids is known to alter the various physical, optical, and thermal properties that help make them applicable for different purposes in automobiles [1,2], solar thermal systems [3,4], refrigeration [5,6], electronics cooling [7], industrial heat transfer systems [8,9], environmental remediation [10], medicine [11,12], etc. The properties of base fluids are influenced by the type, size, shape, and composition of the nanomaterial contained in it [13,14]. Such dispersions, commonly known as nanofluids, have gained immense importance as heat transfer fluids. Several studies reveal their intensifying performance on heat transfer systems [15,16]. Nanofluids exhibit high thermal transport because of the high thermal conductivity of the nanoparticles [17]. Various mechanisms have been identified that play a role in imparting high thermal transport properties by the addition of nanoparticles in liquids [18]; however, these mechanisms are also impacted by various factors related to the nanomaterial type and its synthesis process, nanoparticle shape and size, concentration of nanoparticles and surfactants, method of nanofluid preparation, temperature, etc. Nanoparticles differ in materials and so do their thermal conductivities. Moreover, their morphology, size, shape, structure, etc., play an important role in determining their thermal properties. As nanotechnology offers a varying range of synthesis techniques, each technique has its effect on the properties and formation of nanomaterials [19]. Studies have widely demonstrated how concentration and temperature of nanofluids influence their various properties [20]. In addition, the use of surfactants is known to alter the nanofluid properties.

One of the important aspects, which also leaves an impact on the nanofluids properties, is the preparation method. Basically, a nanofluid can be prepared either by a one-step method or a two-step method. In the one-step method, the nanomaterial synthesis and nanofluids preparation are combined, i.e., the nanoparticle synthesis is directly conducted using the base fluid as the medium and is used for further applications. In the two-step method, the nanofluid is prepared after the nanomaterial is synthesized in a separate synthesis protocol, i.e., the dried nanomaterial particles are dispersed in the base fluid using a mixing or homogenizing technique [21]; however, this mixing of nanomaterial into the base fluid is not as straightforward as it seems. As known from the research on nanofluids, the more the nanoparticles are dispersed, the better suspension stability they acquire [22]. Nanoparticles are anticipated to be evenly and uniformly distributed in the nanofluid to ensure better performance. Clustering or settling of nanoparticles deteriorate properties of nanofluids, demeaning them from being known as colloids. At the same time, the thermophysical properties are directly influenced by the nanofluid stability [23]. Nanofluids, for the purpose of heat transfer, have gained importance because of their high thermal conductivity. Thermal conductivity is an important measure of the heat transfer ability of a nanofluid. Furthermore, settling, aggregation, and clustering of nanoparticles within the nanofluids are a threat to its thermal conductivity.

Ultrasonication is one of the widely used techniques for nanomaterial dispersion in a base fluid [24,25,26]. It involves application of high-frequency sound waves to bring about pressure changes in the fluid. These changes in pressure induce a cavitational effect. Cavitation is a phenomenon in which the pressure differences cause the generation of cavities, which grow in size and collapse after a certain time, releasing a great amount of energy in the form of heat [27]. The temperature and pressure conditions achieved due to cavitation can reach up to 10,000 K and 1000 atm at the micro-level [28]. Such conditions help the breaking of the nanoparticle clusters into tiny nanoparticles and thus dispersing them in the medium [29]; thus, this technique is very useful in nanofluid preparation; however, there are still uncertainties regarding the use of ultrasonication to bring desired changes in the nanofluid properties. Since the wide use of graphene-based nanomaterials for nanofluid application [30], Sandhya et al. [31] presented an extensive review of the effect of ultrasonication for the preparation of graphene nanofluids. Still, the behavior of different nanomaterials under the influence of ultrasonication is not eminent. Ultrasonication involves localized high temperature–pressure conditions, which may alter the physical, as well as chemical, structure of nanomaterials and ultimately influence the nanofluid properties [32]. All of these aspects remain as a gap in the study of nanofluids. Another major aspect is the duration for which ultrasonication must be carried out to achieve an improvement in the thermal properties. It is found that the thermal conductivity of nanofluid increases with an increase in ultrasonication time [33]. The high amplitude of sonication power leads to low aggregate size, high zeta potential, better particle dispersion, and lesser optimum duration [34] (optimum sonication duration for nanofluids is lesser at a high amplitude of sonication power than that achieved for sonication performed at a lower amplitude of sonication power). Mahbubul et al. [35] found that an optimum ultrasonication duration of 150 min is suitable for titania nanofluids. It was also claimed that the average cluster size and ultrasonication time have an interaction effect on zeta potential. In addition, pH is known to be affected by ultrasonication time, thereby affecting the nanofluid stability. Further, Asadi et al. [36] found that multi-walled carbon-nanotube-based nanofluids of different concentrations (0.1, 0.3, and 0.5 vol.%) exhibit maximum thermal conductivity (0.622, 0.638, and 0.66 W m${}^{-1}$K${}^{-1}$) and stability after a ultrasonication period of 60 min. Similarly, the nanofluid also exhibits minimum viscosity due to uniformly distributed nanoparticles [37]. Xian et al. [38] found that an ultrasonication period of 90 min produced highly stable hybrid nanofluid containing mixture of carboxyl-functionalized graphene nanoplatelets and TiO${}_{2}$ nanoparticles with addition of surfactants. Zheng et al. [39] demonstrated that, for very low concentrations of liquid paraffin-based Fe${}_{3}$O${}_{4}$ nanofluid (0.005–0.03 vol.%), the optimum duration of sonication is 3 h. The summary of studies on the influence of the sonication process on the physical properties can be found in recent review paper presented by Asadi et al. [40].

In this work, the influence of ultrasonication time on thermal conductivity of Fe${}_{3}$O${}_{4}$ nanofluid is investigated. For this, water-based nanofluids having different concentrations of Fe${}_{3}$O${}_{4}$ nanoparticles were prepared using bath ultrasonication. Further, the thermal conductivity of these nanofluids was measured at ultrasonication duration from 10 to 55 min.

## 2. Materials and Methods

In this section, a description of the materials used in the study along with the methodology is presented.

### 2.1. Materials

Anhydrous FeCl${}_{3}$ (96% purity) and FeSO${}_{4}$·7H${}_{2}$O (99% purity) purchased from Merck Specialities Pvt Ltd., Mumbai, India and Loba Chemie Pvt Ltd., Mumbai, India, respectively, were used as precursors to synthesize Fe${}_{3}$O${}_{4}$ nanoparticles. In addition, NaOH (98% purity) was used as a precipitating agent, which was procured from Loba Chemie Pvt Ltd., Mumbai, India. Distilled water was used for preparation of solutions, nanofluids, and washing purposes.

### 2.2. Synthesis of Fe${}_{3}$O${}_{4}$ Nanoparticles

Several different methods for the preparation of magnetic nanoparticles have appeared [41,42]; however, in this study, we have used the ultrasound-assisted co-precipitation method to synthesize Fe${}_{3}$O${}_{4}$ nanoparticles. For this, 50 mL solutions containing 0.278 g FeSO${}_{4}$·7H${}_{2}$O and 0.324 g FeCl${}_{3}$ were prepared and sonicated for 5 min. Sonication was continued for another 25 min, wherein the precipitation of Fe${}_{3}$O${}_{4}$ nanoparticles was accomplished due to rise in pH to 11 by drop-by-drop addition of 1 M NaOH solution. The ultrasonicator used was a bath-type ultrasonicator (Dakshin Ultrasonics) having a fixed frequency of 30 kHz. The product was filtered, washed with distilled water, and dried in an oven for 1 h at 100 °C.

### 2.3. Characterization

The ultraviolet-visible (UV-vis) spectrum of the synthesized Fe${}_{3}$O${}_{4}$ nanoparticles was recorded on UV-vis spectrophotometer (LABINDIA Analytical UV3200 model). The transmission electron microscope (TEM) image of the sample was taken using JEOL JEM-1400 Flash Electron Microscope. Fourier transform infrared spectroscopy (FTIR) spectrum of the Fe${}_{3}$O${}_{4}$ nanoparticles was obtained using the Bruker Alpha II instrument. Finally, the X-ray diffraction (XRD) pattern of the Fe${}_{3}$O${}_{4}$ nanoparticles was obtained using Rigaku’s Miniflex 1800 diffractometer.

### 2.4. Synthesis of the Fe${}_{3}$O${}_{4}$ Nanofluids

Water-based nanofluids containing different concentrations of Fe${}_{3}$O${}_{4}$ nanoparticles (in two fractions of nanoparticles: 0.01, 0.025 vol.%) were prepared by dispersing the nanoparticles using ultrasonication in a bath ultrasonicator (Dakshin Ultrasonics) working at a fixed frequency and power of 30 kHz and 500 W, respectively. The bath had a capacity of 30 L. The nanofluids were initially ultrasonicated for 10 min in strictly controlled temperature.

### 2.5. Thermal Conductivity Measurement

The thermal conductivity of the Fe${}_{3}$O${}_{4}$ nanofluid in the examined concentrations was measured using the KD2 Pro thermal property analyzer (Decagon Devices Inc., Pullman, Washington, DC, USA). To determine the effect of ultrasonication time, the thermal conductivity of the nanofluid was measured at intervals of 5–10 min within the total ultrasonication time of 55 min conducted in the bath ultrasonicator. Temperature was maintained at 303.15 K for all the measurements.

#### Uncertainty Determination

**Figure 1:**
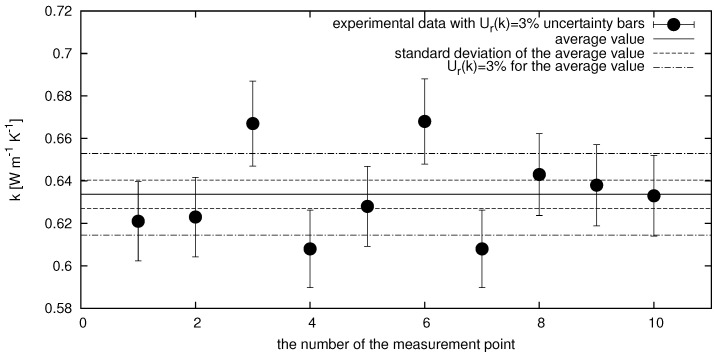
Results of the ten consecutive measurements of the thermal conductivity of water performed at 303.15 K. Points refers to experimental data; solid lines present average value of performed series of experiments; dotted lines show the standard deviation of the average and relative uncertainty of 3% of the average.

The uncertainty of the results obtained with the above-described measuring device was determined by performing a series of measurements of the thermal conductivity of water and then determining the standard deviation of the results. Ten consecutive measurements of the thermal conductivity of water were performed at 303.15 K. The results of these measurements are summarized in Figure 1. The value of the thermal conductivity of water determined with this measurement was k=0.6337 W m${}^{-1}$K${}^{-1}$ with the standard deviation u(k)=0.0067 W m${}^{-1}$K${}^{-1}$, which is 1.1%. Taking into account this result, the expended relative uncertainty (with K=2) was determined to be 3% of the experimental value, which corresponds with the value presented in the literature [43,44,45]. Finally, the obtained result of the thermal conductivity of water k=(0.634±0.020) W m${}^{-1}$K${}^{-1}$ is in good agreement with the literature data presented by Shokouhi et al. [46] as k=(0.613±0.002) W m${}^{-1}$K${}^{-1}$.

## 3. Results and Discussion

In this section, the characterization of developed nanofluids is presented. The results of the study are summarized along with the discussion of mechanisms that leads to observed behavior.

### 3.1. Formation of Fe${}_{3}$O${}_{4}$ Nanoparticles and Its Characterization

**Figure 2:**
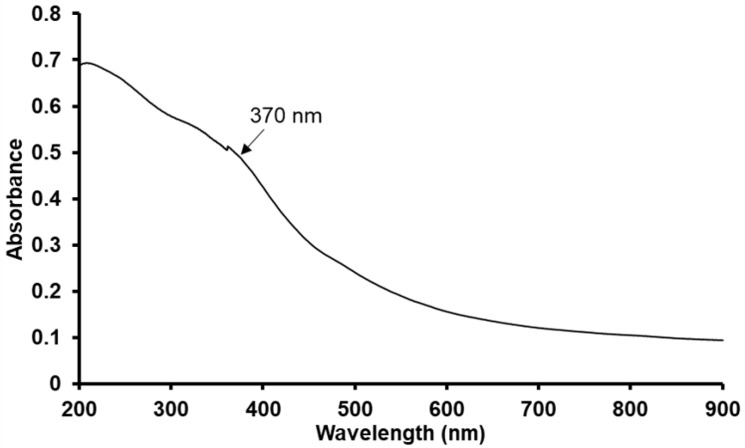
Ultraviolet-visible (UV-vis) spectrum of the synthesized Fe${}_{3}$O${}_{4}$ nanoparticles.

The formation of Fe${}_{3}$O${}_{4}$ nanoparticles was confirmed using various characterization techniques. Figure 2 shows the UV-visible spectra of synthesized Fe${}_{3}$O${}_{4}$ nanoparticles. A small peak at 370 nm and the absorption edge between 375 and 650 nm confirms the successful formation of Fe${}_{3}$O${}_{4}$ nanoparticles [47]. The origin of the decaying absorption tail after 200 nm up to 900 nm is attributed to the fact that the material does not absorb any radiation in the visible range of the electromagnetic spectrum.

**Figure 3:**
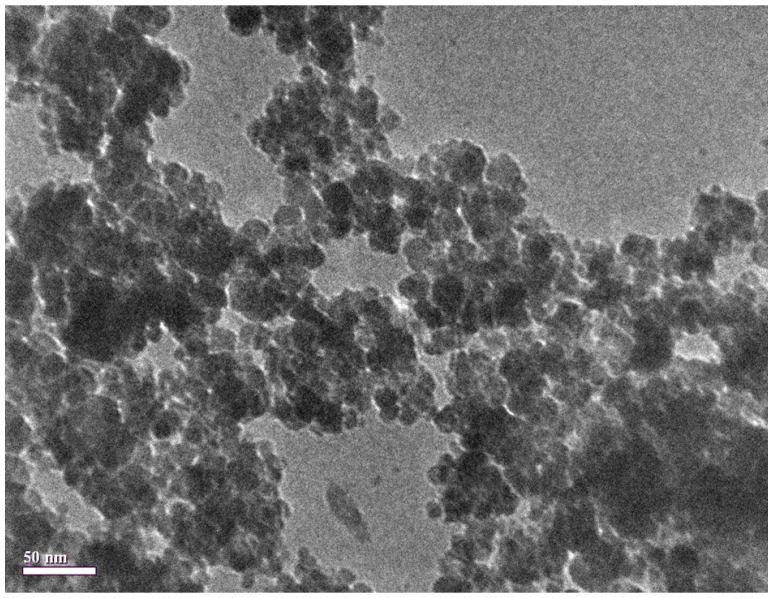
Transmission electron microscope (TEM) image of synthesized Fe${}_{3}$O${}_{4}$ nanoparticles.

Figure 3 shows the TEM image of synthesized Fe${}_{3}$O${}_{4}$ nanoparticles. The image shows that the Fe${}_{3}$O${}_{4}$ nanoparticles have a spherical shape and polydisperse nature. The particle size as observed from the TEM image ranges between 6 to 21 nm; however, the average particle size of the Fe${}_{3}$O${}_{4}$ nanoparticles is estimated to be around 11.7 nm.

**Figure 4:**
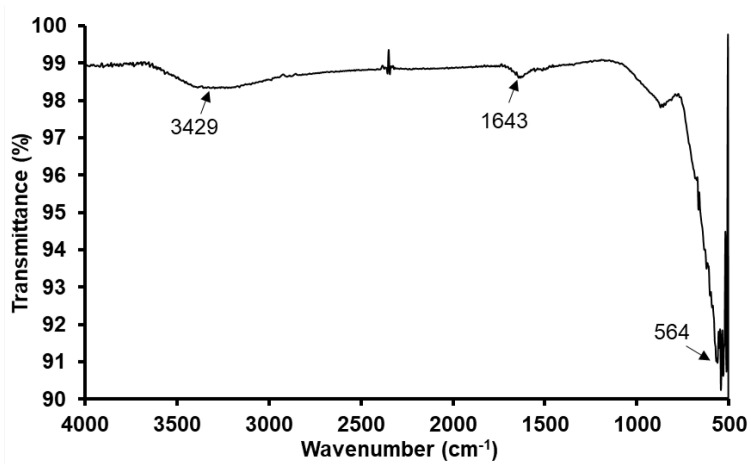
Fourier transform infrared spectroscopy (FTIR) spectra of synthesized Fe${}_{3}$O${}_{4}$ nanoparticles.

Figure 4 depicts the FTIR spectra of synthesized Fe${}_{3}$O${}_{4}$ nanoparticles. It shows strong absorption between 550 cm${}^{-1}$ to 680 cm${}^{-1}$. The most intense peak at 564 cm${}^{-1}$ represents the Fe-O bond within the Fe${}_{3}$O${}_{4}$ structure [48,49]. Further, the band at 1643 cm${}^{-1}$ is attributed to the OH-bending and the one at 3429 cm${}^{-1}$ is attributed to the OH-stretching. These bands occur due to the presence of hydroxyl groups.

**Figure 5:**
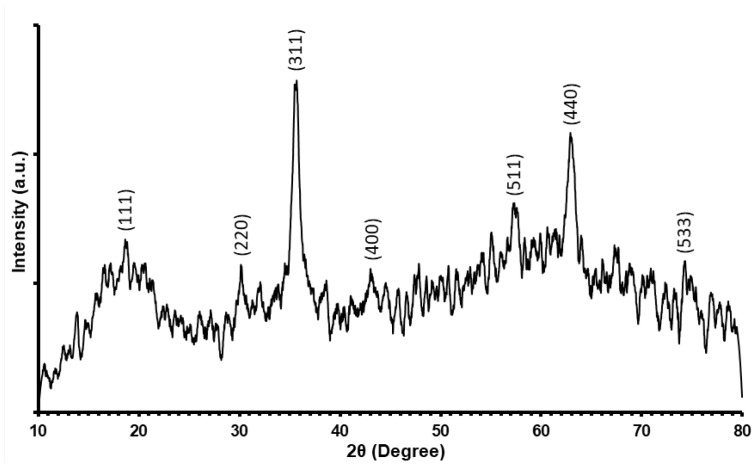
X-ray diffraction (XRD) pattern of synthesized Fe${}_{3}$O${}_{4}$ nanoparticles.

The XRD pattern of the synthesized Fe${}_{3}$O${}_{4}$ nanoparticles is depicted in Figure 5. The peaks at 18.6°, 30.1°, 35.6°, 43°, 57.3°, 62.9°, and 74.3° represent the planes of Fe${}_{3}$O${}_{4}$ at (111), (220), (311), (400), (511), (440), and (533), respectively, confirming its cubic spinel phase [50]. The average crystallite size obtained from Debye–Scherrer equation based on the most intense XRD peak is estimated to be 25.97 nm. In addition, the lattice constant for the Fe${}_{3}$O${}_{4}$ spinel nanoparticles is found to be 8.34(3) Å, which is in accordance with that available in literature [51].

### 3.2. The Effect of Ultrasonication Time on Thermal Conductivity

**Figure 6:**
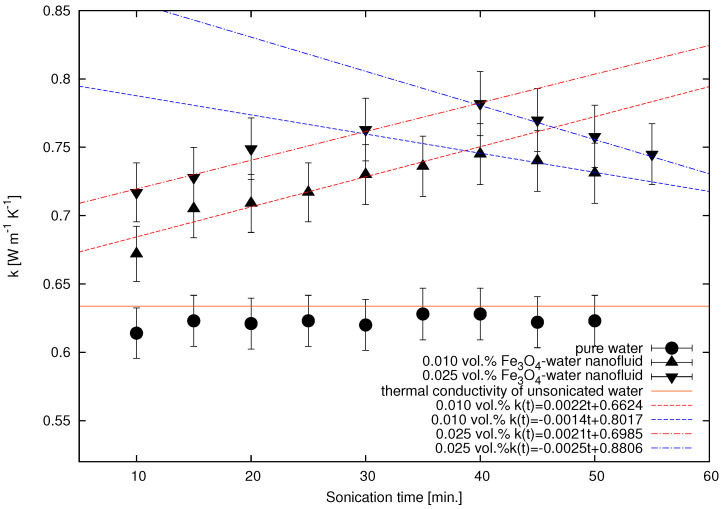
Thermal conductivity of pure water and Fe${}_{3}$O${}_{4}$ nanofluid at different concentrations as a function of ultrasonication time. Symbols present experimental data; solid line shows the value of thermal conductivity of unsonicated water; dotted lines present linear increase and decrease in thermal conductivity during sonication.

Figure 6 shows the trend of thermal conductivity of water and different concentrations of Fe${}_{3}$O${}_{4}$ nanofluid with respect to the ultrasonication time. As usual, the thermal conductivity of water is almost constant with respect to time even after ultrasonicating for the previously mentioned duration.

**Figure 7:**
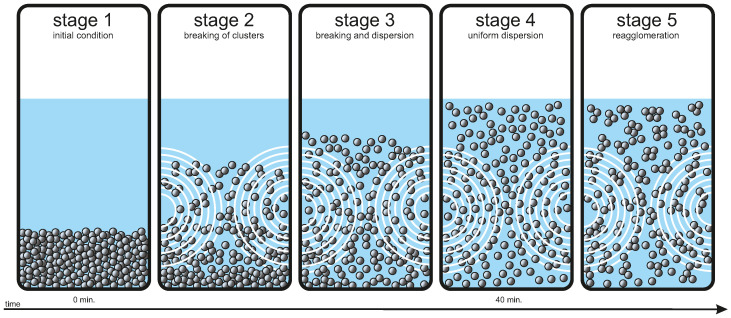
Various stages of nanofluid during ultrasonication.

The thermal conductivity of the nanofluid depends on the ultrasonication time. The term ‘agglomerate’ refers to the weak interaction of the nanoparticles, thereby forming clusters that can be broken by physical forces similar to those of ultrasonication; however, the aggregates composed of particles that are connected together (welded) with solid necks are difficult to break [52,53]. Initially, the nanoparticles added to the base fluid, here water, are in an agglomerated state. The thermal performance of the nanofluid is lesser at higher agglomeration conditions [31]. For the Fe${}_{3}$O${}_{4}$ nanofluid concentrations, i.e., 0.01 and 0.025 vol.%, the thermal conductivity increases with ultrasonication time until 40 min of duration, after which it starts to deteriorate. At a lesser ultrasonication duration, there is insufficient cavitation, which is not able to break the nanoparticle clusters (Stage 1); this causes the fluid to remain unstable and thereby exhibit lesser thermal conductivity. As the ultrasonication duration increases, more and more nanoparticle agglomerates or clusters break, releasing tiny nanoparticles into the nanofluid (Stage 2) [54]. Firstly, the larger clusters are broken down into smaller clusters and then into individual nanoparticles [55]. Further increase in ultrasonication duration not only contributes to the breaking down of the clusters, but also to disperse the tiny nanoparticles uniformly throughout the nanofluid body (Stage 3) [55]. This process continues until the ultrasonication duration of 40 min ends. The evenly dispersed nanoparticles aid in imparting high thermal conductivity to the fluid due to the participation of the maximum number of nanoparticles in various heat transfer mechanisms (Stage 4); however, the decrease in thermal conductivity after 40 min is an indication of the reagglomeration of the nanoparticles into clusters, thereby again decreasing the nanofluid stability. This is caused by the excessive Brownian motion and enhanced contact between individual nanoparticles that lead to their interaction and thus increased clustering [56]. The clustering of nanoparticles gives rise to settling and thus the deterioration of the nanofluid stability. As stability is directly related to the nanofluid thermal conductivity, unstable nanofluids exhibit worse thermal conductivity [36]. All these stages are graphically represented in Figure 7. The linear increase and decrease in the thermal conductivity value during the sonication time can be modeled with simple functions as presented in Figure 6.

Furthermore, thermal conductivity of 0.025 vol.% Fe${}_{3}$O${}_{4}$ nanofluid was recorded to be 0.717 W m${}^{-1}$K${}^{-1}$, which was more than that of 0.01 vol.% Fe${}_{3}$O${}_{4}$ nanofluid (0.672 W m${}^{-1}$K${}^{-1}$). This is consistent at all instances of ultrasonication time. This is due to the larger number of nanoparticles, as heat carriers, taking part in thermal transport [20]. At higher concentrations, a greater number of nanoparticles, having their characteristic Brownian motion, efficiently transfer heat energy due to the induced micro-movement.

## 4. Conclusions

This work demonstrates the effect of ultrasonication at various durations (10 to 55 min) on the thermal conductivity of the Fe${}_{3}$O${}_{4}$ nanofluid. It was found that the optimum duration of ultrasonication for water-based Fe${}_{3}$O${}_{4}$ nanofluid is 40 min, at which it shows maximum thermal conductivity. After 40 min of ultrasonication, the thermal conductivity starts to deteriorate. For a 0.025 vol.% of Fe${}_{3}$O${}_{4}$ nanoparticles in the nanofluid, the thermal conductivity increased from 0.717 W m${}^{-1}$K${}^{-1}$ to 0.782 W m${}^{-1}$K${}^{-1}$ with an increase in ultrasonication time from 10 min to 40 min. This is due to the breaking of the clusters of the nanoparticles due to ultrasonication; however, the thermal conductivity further deteriorated to 0.745 W m${}^{-1}$K${}^{-1}$ after an ultrasonication time of 40 min up to 55 min. This is due to the excessive Brownian-motion-induced contact and clustering, which further lead to settling and the decrease in the stability of the nanofluid. 

## Data Availability

Not applicable.

## Abbreviations

UV-vis	Ultraviolet-visible
TEM	Transmission electron microscope
FTIR	Fourier transform infrared spectroscopy
XRD	X-ray diffraction
